# Single-Molecule Monitoring of Nucleic Acid Dynamics Using Raman Correlation Spectroscopy in Plasmonic Nanowells

**DOI:** 10.1021/acsnano.5c12000

**Published:** 2025-10-14

**Authors:** Peilin Xin, Yingqi Zhao, Yuge Liang, Mulusew W. Yaltaye, Aliaksandr Hubarevich, Viktorija Pankratova, Shubo Wang, Jian-An Huang

**Affiliations:** † Research Unit of Health Sciences and Technology, Faculty of Medicine, 6370 University of Oulu, Aapistie 5 A, 90220 Oulu, Finland; ‡ Research Unit of Disease Networks, Faculty of Biochemistry and Molecular Medicine, University of Oulu, Aapistie 5 A, 90220 Oulu, Finland; § Biocenter Oulu, University of Oulu, Aapistie 5 A, 90220 Oulu, Finland; ∥ 121451 Istituto Italiano di Tecnologia, Via Morego 30, 16163 Genova, Italy; ⊥ Nano and Molecular Systems Research Unit, Faculty of Science, University of Oulu, Pentti Kaiteran katu 1, 90570 Oulu, Finland

**Keywords:** single-molecule monitoring, SERS, plasmonic hot spot, Raman correlation spectroscopy, citrate interference

## Abstract

Label-free monitoring of single molecules by single-molecule surface-enhanced Raman spectroscopy (SM-SERS) in plasmonic nanopores can track the molecular dynamics and gain insight into its internal mechanism for applications including catalysis and sequencing. However, challenges including unstable plasmonic hot spot, fast molecule movement, and citrate interference hinder the SM-SERS data analysis and biomedical applications. In this study, we report a new SM-SERS method by sticking a single gold nanoparticle in a gold nanowell in air to generate a fixed plasmonic gap-mode hot spot on the particle surface for continuous single-molecule readout and long-term monitoring of DNA diffusion. The unlimited resident time of the DNA in the hot spot revealed unidirectional and back-and-forth diffusion patterns of different DNAs at single-base resolution depending on their sequences as well as cooccupation of the hot spot by citrate and DNA. Significantly, the spatial resolution of the hot spot was found to be able to cover 2 neighboring nucleobases, 1 sugar–phosphate backbone in the DNA, and 1 citrate. By using Raman correlation spectroscopy, the diffusion times of nucleobases in the DNAs were calculated as 5–22 s depending on molecular structures, while those of citrate were 0.1–7 s. Our results were so promising for monitoring biomolecular dynamics that they could be used to investigate oligonucleotide hybridization kinetics and may set the basis for developing SM-SERS sequencing technologies.

## Graphical abstract

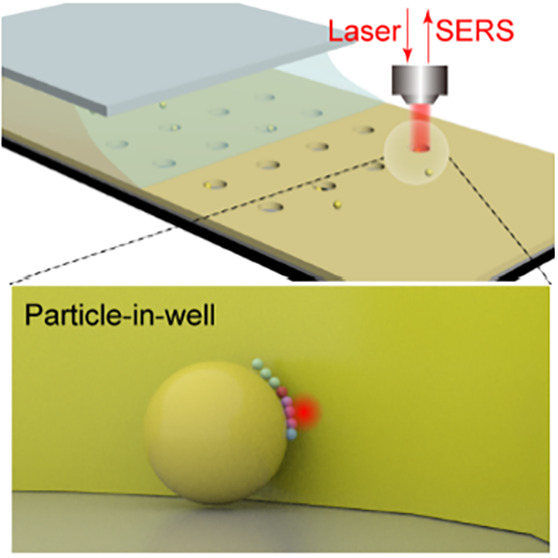

Label-free monitoring of single molecules can uncover traits that are hard to detect in molecular assemblies. It allows real-time tracking of a single molecule’s changes under specific conditions and provides insight into the internal mechanisms of molecular dynamics that can improve our understanding of complex chemical and biological processes. As an important label-free method, single-molecule surface-enhanced Raman scattering (SM-SERS) constructed a localized and amplified electromagnetic field, so-called plasmonic hot spot, to enhance and detect the intrinsic Raman scattering signals of single molecules with high spatial and spectral resolutions for understanding catalysis, chemical reactions, and quantum optics.^1–3^

Recent SM-SERS methods based on solid-state plasmonic nanopores and nanocavities are emerging and promising sequencing techniques for both DNAs and proteins due to their successful discrimination of 4 DNA bases and 20 proteogenic amino acids.^4^ They could pave the way for single-molecule protein sequencing that aims to identify the amino acid residues in a single protein and determine their order without the need of protein amplification.^5^ For example, Chen et al. used an engineered elongated nanopore structure to absorb DNA analytes temporarily inside the pores and observed asynchronous spectroscopic behavior of different nucleobases in DNA strands.^6^ Kang Wang’s group used gold plasmonic nanopores to drive the transfer of a single DNA oligonucleotide or protein through nanopores by applying a bias potential, thereby collecting rich biomolecular structural information through SM-SERS sequencing.^7^ To overcome the irregular surface charge of different amino acid residues in proteins, DNAs were added at the protein terminus for electroosmotic translocation of the DNA–protein–DNA construct through nanopores by electric bias.^8^ However, the insufficient temporal resolutions of the plasmonic nanopores face challenges to match the fast translocation of the biomolecules through the hot spot.^4^ For example, the plasmonic bowl-shaped nanopore generated electroosmotic shear flow under electric bias to translocate DNA through the hot spot of the nanopore. But the movement of the DNA segment through the hot spot was not consecutive but, instead, in a jump-and-attach manner.

Against the fast electrically driven translocation of biomolecules through the nanopores, SM-SERS monitoring of the Brownian diffusion of biomolecules through the hot spot by dynamic SERS was explored for sequencing.^9,10^ For example, our group studied the SM-SERS monitoring of biomolecules by the plasmonic Particle-in-Pore platform that adsorbed biomolecules on a gold nanoparticle and then trapped the particle into a gold nanopore in liquid for tens of seconds. The particle trapping generated a single hot spot on the nanoparticle surface that allowed SM-SERS monitoring of the diffusion of a single peptide at single-amino-acid resolution and the peptide conformation changes during its diffusion.^11^ Despite the single-amino-acid resolution, the trapped nanoparticle experienced Brownian movement in the nanopore such that the excitation area on the molecule by the hot spot was continuously changing. The resultant SM-SERS signal fluctuations were affected by dynamics of both particle and molecules.^12^ Moreover, the presence and diffusion of citrate on the nanoparticle surface generated a dynamic SM-SERS background that interfered with the SM-SERS signals of analyte molecules.^13^ All of these presented challenges in data analysis of the SM-SERS signals and sequence reconstruction.

Meanwhile, the short monitoring time of the Particle-in-Pore sensor hinders their applications to monitoring of nuclear acid dynamics for pathological investigation and therapeutic development.^12^ For example, microRNAs are responsible for post-transcriptional gene expression regulation and biomarkers of many human diseases, including cancers and therapeutic development. However, label-free detection of microRNA suffers from their short sequence, low abundance, and high similarity among family members at a single-base difference.^14^ The Particle-in-Pore sensor capable of monitoring single DNA with single-base resolution and temporal resolution of 0.1 s shows the potential for SM-SERS monitoring of the oligonucleotide hybridization kinetics of microRNAs. Nevertheless, the changing hot spot in the Particle-in-Pore sensor limited its monitoring time to be shorter than that required for microRNA hybridization with DNA.^15,16^

Here, we present a new plasmonic Particle-in-well sensor that fixed the nanoparticle near the gold nanowell sidewall by van der Waals force and generated a static hot spot for SM-SERS monitoring of DNA dynamics at single-base resolution for unlimited time.^17^ First, the DNA oligonucleotide was physically absorbed on the surface of the gold nanoparticles (AuNPs) in PBS buffer. Then, the nanowell can be made by Focused Ion Beam (FIB) after the gold layer. The AuNPs, carrying analytes, were entrapped into the gold nanowell system and dried via the directed capillary-assisted particle assembly (CAPA) method (Figure 1A). Once captured within the nanowell, the particles remain permanently confined, thereby enabling a continuous signal readout and ensuring long-term operational stability. The resulted nanogap between the nanoparticle and the nanowell generated a gap-mode plasmonic hot spot for SM-SERS biomolecule monitoring, providing insights into molecular diffusion (Figure 1B) and movement trend in the hot spot (Figure 1C).

**Figure 1:**
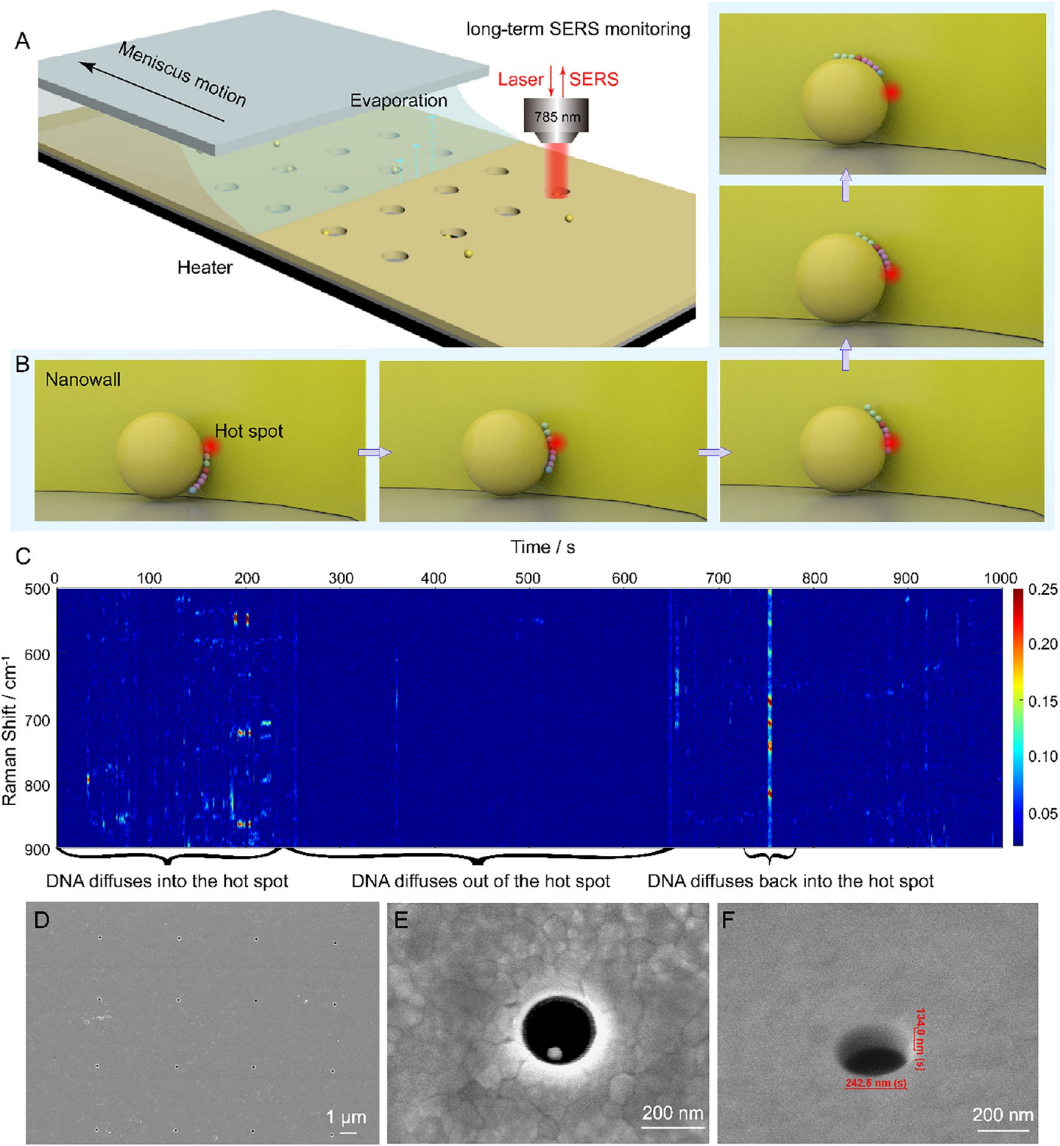
Fabrication of the Particle-in-well sensor and the SERS detection of single-molecule movement in its hot spot. (A) Entrapment of the DNA-loaded AuNP in the gold nanowell via the directed capillary assembly method. (B) The unidirectional DNA oligonucleotide diffusion in the hot spot on the AuNP. (C) A 17 min waterfall plot of the SERS time series of a DNA oligonucleotide with three stages: (1) DNA diffuses into the hot spot; (2) DNA diffuses out of the hot spot, and (3) DNA diffuses back into the hot spot. (D) SEM image of a nanowell array spaced more than 4 μm apart with an FIB drilled nanowell on a 100 nm Au layer on a Si_3_N_4_ wafer. (E) The top-view SEM image of the AuNP trapped in nanowell. (F) The SEM image of the nanowell with depth and height.

For the temporal characterization, the Particle-in-well sensor was combined with single-molecule Raman correlation spectroscopy (RCS). RCS is based on the analysis of time correlations in Raman signal fluctuation emitted when the molecules or particles are diffusing in and out of the hot spot, such as observing the particle diffusion motion or molecule diffusion in solution by Raman signal.^18,19^ As an application demonstration, we used RCS to calculate the diffusion times of analyte and citrate molecules on the surface of the nanoparticle and mitigate citrate interference by comparing their respective frequencies and diffusion times. Our method is capable of discriminating single molecules by their diffusion times and monitoring long-term movement by entrapping and detecting single bases from oligonucleotide fragments using time-resolved SM-SERS spectra and the RCS technique.

## Results and Discussion

### Fabrication and Characterization of Particle-in-Well Sensor

To fabricate the Particle-in-well platform, we first sputtered a 100 nm thick gold layer onto the surface of a Si_3_N_4_ wafer with dimensions of 2 cm × 1 cm. Nanowell arrays consisting of 30 subarrays (4 × 4) were subsequently fabricated on the 100 nm thick gold film using FIB technology, as depicted in Figure 1D and E. The nanowell had a diameter of around 242 nm and a depth of around 134 nm (Figure 1F), as measured by atomic force microscopy (AFM) (Figure S1, Supporting Information). The AFM result matched well with those obtained from the result of scanning electron microscopy (SEM), further verifying the nanowells’ dimensions.

To realize AuNPs entrapment, we first adsorbed the DNA oligonucleotide on the AuNPs and then trapped single DNA-loaded AuNPs into the nanowells using the CAPA method (see METHODS for details) as described in Figure 1A.^20–22^ The working principle of CAPA is to move the meniscus of an aqueous colloidal suspension droplet onto a topographic template, and the capillary force of the meniscus places the particles at a designated location. The packing of stable nanoparticles depends on the properties of the colloidal solution, surface tension, surfactant concentration, and interparticle interactions. In our study, CAPA allowed the placement of AuNPs in the nanowells. What’s more, the AuNPs will not aggregate after DNA oligonucleotide absorption by dynamic light scattering (DLS) verification (Figure S2, Supporting Information). After entrapment of DNA-loaded AuNPs, the long-term SERS detection can be realized in the Particle-in-well sensor in Figure 1C. Once the AuNPs with adsorbed molecules were trapped, they were permanently stuck with the nanowell sidewall with salt residue between them to form the gap-mode plasmonic hot spot. As shown in Figure S3, after 40 min of testing and 6 months of storage, the DNA-loaded AuNPs are still trapped in the nanowell, which proved the permanent confinement of the nanoparticle in the nanowell. This ensures the long-term stability of the hot spot by preventing undesired particle displacement during applications. This Particle-in-well sensor allows for excellent control over the structural parameters of the nanowells, ensuring uniform quality.

### Validation of Single-Molecule Sensitivity

To validate single-molecule SERS, the bianalyte SERS (BIASERS) technique was employed to detect pyridine and its deuterated analogue, pyridine-d5, verifying the single-molecule sensitivity of the Particle-in-well platform. Pyridine and pyridine-d5 have the same chemical properties, with deuterium atoms substituting for the hydrogen atom on the pyridine ring. Due to the replacement of deuterium, the SERS peak of pyridine was shifted from 990 to 960 cm^–1^.^23^ The submonolayer of analyte consisted of equal numbers of pyridine and pyridine-d5 was absorbed on the gold surface. The SERS signal was collected under 785 nm laser with 18–22 mW and 0.1 s accumulation time, which strikes a balance between fast spectral collection and sufficient signal for data analysis. Improvement of signal intensity can be easily achieved by prolonging the accumulation time; however, it will decrease the temporal resolution of monitoring DNA dynamics. As shown in Figure 2A, the time series SERS signals of 2000 spectra of pyridine and pyridine-d5 were acquired and analyzed from one trapping event in one hot spot. All spectra have been preprocessed using the MATLAB program (see METHODS for details). The majority of signals were confined between 900 cm^–1^ and 1000 cm^–1^. The spectral fluctuation is typical for single-molecule SERS, which resulted from the Brownian diffusion of these molecules on the gold surface. As shown in Figure 2B, the SERS peak in the 950–970 cm^–1^ range corresponds to single pyridine-d5 in the hot spot, whereas the one in the 980–1000 cm^–1^ region is assigned to single pyridine in the hot spot. The appearance of both peaks indicates the existence of both pyridine and pyridine-d5 in the hot spot.

**Figure 2:**
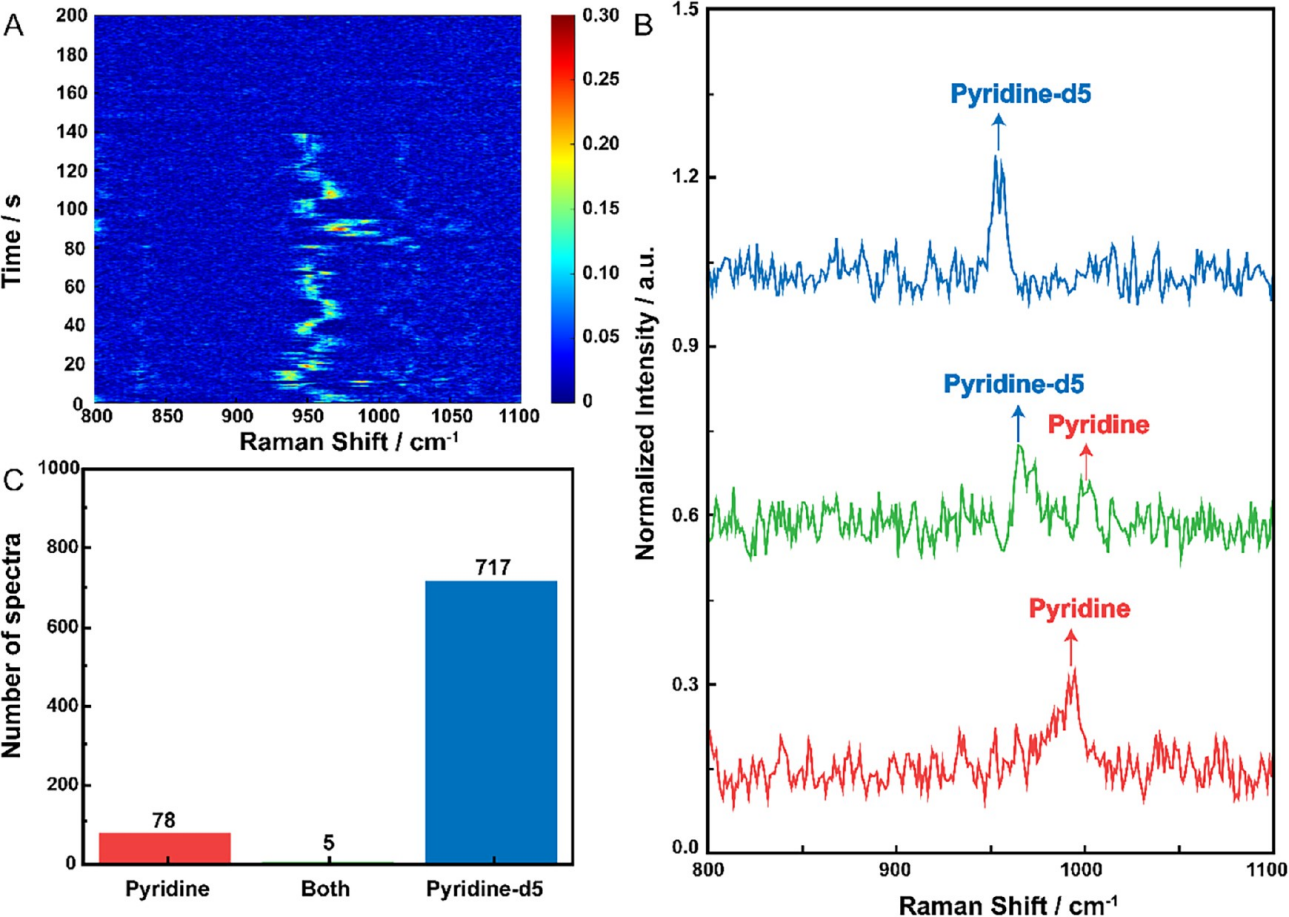
BIASERS single-molecule detection for pyridine and pyridine-d5. (A) A waterfall plot of the SERS time series of pyridine and pyridine-d5. (B) SERS spectrum comparison between pyridine (with 995 cm^–1^ peak), pyridine-d5 (with 954 cm^–1^ peak), and both. (C) The frequency histograms were for pyridine, pyridine-d5, and both.

For a conventional BIASERS test with sufficient data collected from a large number of hot spots, the single-molecule-event numbers of both molecules will tend to be comparable. However, here in the Particle-in-well sensor, the BIASERS data are collected from one static hot spot in around 3 min and 20 s. It shows the random diffusion of pyridine and pyridine-d5 in, from 0 s, and out of the hot spot at 140 s. Considering the limited number of spectra, a comparable number of spectra for both molecules has not been reached. However, the most important criterion for determining single-molecule detection is the negligible number of two-molecule spectra compared with the single-molecule spectra. As summarized in Figure 2C, there were 717 events from the single pyridine-d5, 78 events from the single pyridine, and only 5 events from both molecules. Notably, only five spectra were identified as containing peaks for both pyridine and pyridine-d5, indicating minimal signal overlap, thus affirming the single-molecule detection capability of this sensor. Therefore, the single-molecule events dominated the SERS signal. The BIASERS results demonstrate that the hot spot size is small enough to cover only one molecule, thus enabling single-molecule detection in the Particle-in-well sensor.

### Characterization of the Citrate Substitution

Interference from citrate has been recognized as a potential problem in single-molecule analysis. However, the complete removal of citrate from the surface of AuNPs remains challenging. Citrate is commonly employed as stabilizers and reducing agents in AuNPs fabrication to guarantee long-term stability.^24^ The presence of citrates can interfere with the SERS signals of DNA oligonucleotide in the Particle-in-well sensor of single-molecule sensitivity.^13^ According to previous reports, DNA nucleobases exhibit a stronger binding affinity to the gold surface than citrate (A > C ≥ G > *T* > citrate).^25,26^ As a result, DNA oligonucleotides are capable of displacing citrate molecules on AuNPs through competitive adsorption.

To characterize the substitution, the statistical comparison of citrate and DNA oligonucleotide signals was analyzed. The citrate signal was collected by trapping the as-purchased AuNPs in the nanowells. As a comparison, DNA oligonucleotide molecules (5′-CCCATTTG-3′) were also absorbed on the AuNPs surface for signal collection in the nanowells. The peak occurring frequency of citrate and DNA oligonucleotide was calculated by counting the times of the peak occurred from 3524 and 3695 effective spectra, as shown in Figure 3A,C.^27,28^ Here, effective spectra mean the spectra with SERS peaks based on the peak height, width, and prominence. Without analyte substitution, in Figure 3A,B, citrate shows high peak occurring frequency in 510–530 cm^–1^(π­(COO) vibration), 620–640 cm^–1^(δ­(COO)), and 880–900 cm^–1^(ν­(C–COO)), but there is almost no signal (frequency less than 5) in these three regions after DNA oligonucleotide substitution, as shown in Figure 3D. This obvious decrease of the peak occurring frequency in these three regions clearly indicates that citrate could be substituted by a DNA oligonucleotide. However, this does not guarantee complete citrate substitution, as analyte adsorption on AuNPs is a dynamic process. Based on reported results, complete citrate replacement is unlikely even when the analyte concentration is large enough to form a monolayer.^28^ Furthermore, citrate may form a secondary coating layer through hydrogen bonding on top of the polar molecule such as DNAs, making complete elimination of citrate interference virtually impossible.^29,30^ The signal impact of citrate, *i.e*., its coexistence, was accounted for in subsequent DNA data analysis. After DNA oligonucleotide substitution, as shown in Figure 3C,D, increased peak frequencies were observed in the regions of 771–791 and 792–812 cm^–1^, which agree with the position of characteristic peaks of thymine (T) and cytosine (C). Furthermore, regions of 725–745 and 645–665 cm^–1^, adenine (A) and guanine (G), did not exhibit high frequencies, which aligns with the lower abundance of adenine and guanine in the DNA oligonucleotide sequence (5′-CCCATTTG-3′). These observations were further confirmed by competitive adsorption and coexistence analysis of DNA oligonucleotides and citrate. Similar analysis has been done to ensure the substitution of citrates by pyridine and pyridine-d5 for BIASERS in Figure S4 (Supporting Information).

**Figure 3:**
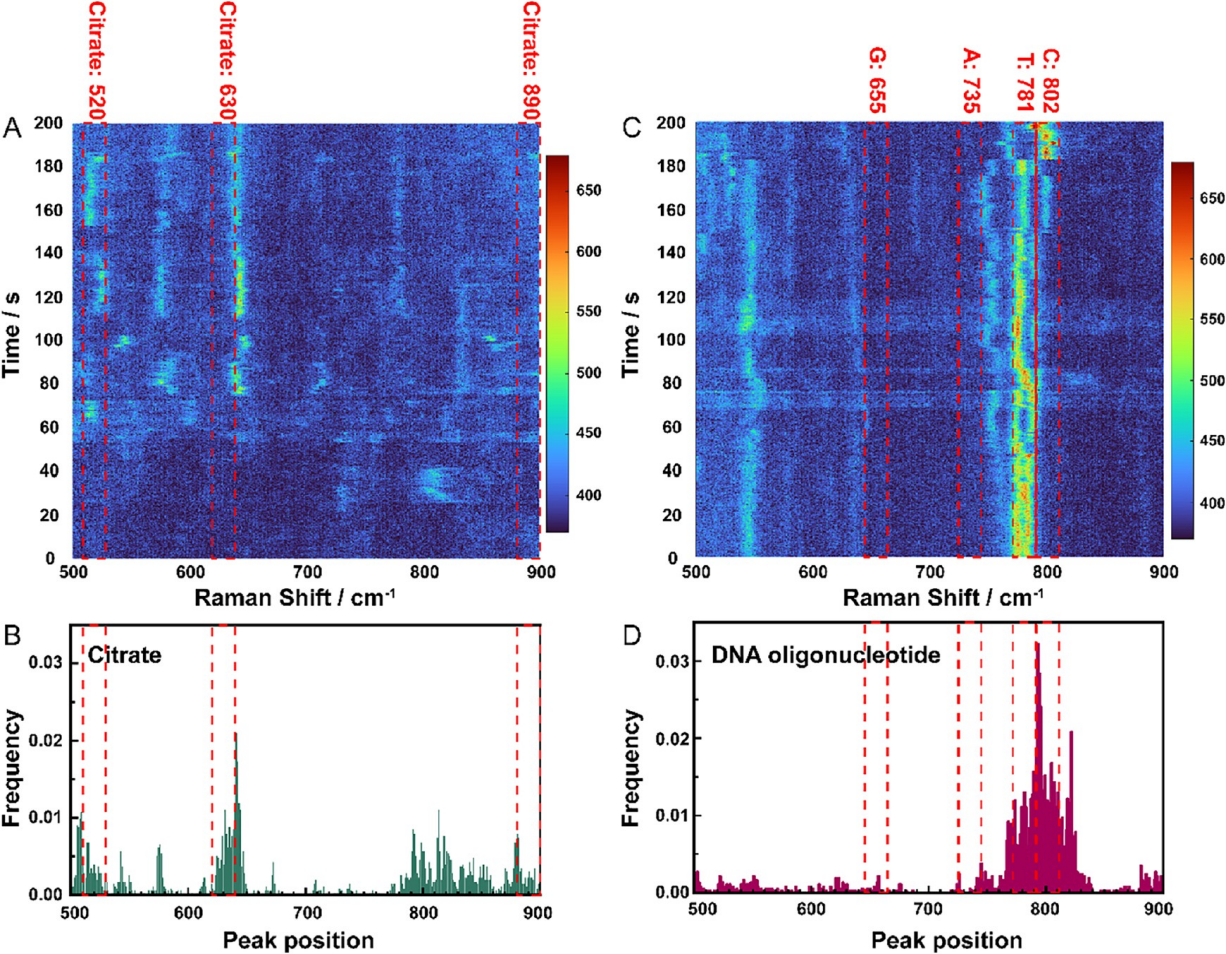
Frequency statistics between citrate and DNA oligonucleotide (5′-CCCATTTG-3′) SERS signal. (A) A waterfall plot of the SERS time series of citrate. (B) The normalized frequency histogram of the citrate SERS signal is 500–900 cm^–1^. (C) A waterfall plot of SERS time series of DNA oligonucleotide. (D) The normalized frequency of the DNA oligonucleotide SERS signal is 500–900 cm^–1^.

### Single-Molecule Monitoring of Neighboring Nucleobase Movement during DNA Diffusion

With single-molecule sensitivity and static hot spot, the Particle-in-well sensor provides the possibility to monitor the dynamic behavior of molecules adsorbed on the AuNPs. The DNA oligonucleotide was used as a model molecule to demonstrate the single nucleobase detection and chain movement monitoring. Similarly, the AuNP with DNA oligonucleotide (15.3 nM) was confined within the nanowell, and SM-SERS spectra were subsequently acquired. To demonstrate single-molecule spectra, 2000 spectra have been collected for extracting the distribution of full width at half-maximum (fwhm) of peaks at 802 and 781 cm^–1^, which indicated the existence of C and T bases, respectively. 72.7% of the C characteristic peak width was below 12 cm^–1^, and the average fwhm was 9.1 cm^–1^. 83.1% of the T characteristic peak showed a width below 12 cm^–1^, and the average fwhm was 8.7 cm^–1^ (as shown in Figure S5, Supporting Information). The narrow fwhm of Raman peaks further confirmed the single-base detection. Our group has reported that the multimolecule peak width is around 15 cm^–1^.^12^

To demonstrate the discrimination of a single nucleobase in a DNA oligonucleotide fragment, 10% coverage of three different sequences in Table 1 was individually adsorbed onto AuNPs via nonspecific interactions between the nucleobases and the gold surface.^31,32^ After adsorption, the DNA-loaded AuNPs were confined in nanowells for signal acquisition. Single-base detection on the DNA oligonucleotide fragment in the Particle-in-well sensor lays the foundation of long-term monitoring of DNA chain dynamics and signal readout at the single-molecule level. Due to specific surface selection rules, nucleotides with different conformations on the gold surface will show different SERS spectra of single DNA nucleobases. Therefore, the conformational information on oligonucleotides can be obtained through the SERS spectra of single nucleobases in oligonucleotides.^33–35^

**Table 1:** Sequences of Three DNA Oligonucleotides Used in the Particle-in-Well Sensor

DNA oligonucleotide No.	Sequence
1	5′-CCCATTTG-3′
2	5′-TAACTGGC-3′
3	5′-TACAAGTAAAG-3′

As illustrated in Figure 4A, the spectra exhibit both peak shifting and temporal continuity, revealing real-time single-molecular behavior. Notably, certain characteristic in-plane ring breathing modes can be significantly suppressed due to the flat-lying molecule configuration under the hot spot, meaning that the configuration of a single molecule within the plasmonic hot spot can dramatically modulate spectral visibility, either enhancing or diminishing even the most intense vibrational modes.^36^ In addition to the peak shift, peak fluctuations are commonly observed. Owing to intrinsic molecular vibrations, the Raman signal from a single nucleobase does not remain fixed but instead displays dynamic drift over time. This temporal spectral movement further highlights the dynamic, conformation-sensitive nature of SM^–^SERS, offering unique access to the motion and spatial orientation of individual DNA nucleobases.^37^ To extract dynamic molecular information, spectra ranging from 168 to 177 s in the same time series data set were selected for analysis as most of the characteristic peaks with minimal fluctuations are in this range. Then, the peak was assigned to the nucleobases according to the corresponding characteristic peaks. As summarized in Table 2, the most characteristic peaks of A, C, G, and T bases are typically located at 735, 802, 655, and 781 cm^–1^, respectively. These peaks correspond to distinct vibrational modes: ring breathing for A, C, and T bases and R6 breathing for the G base. As in SM-SERS spectra, these peak positions fluctuate around their average values. Therefore, a ±10 cm^–1^ window around the characteristic peaks was set for accurate peak assignment.

**Figure 4:**
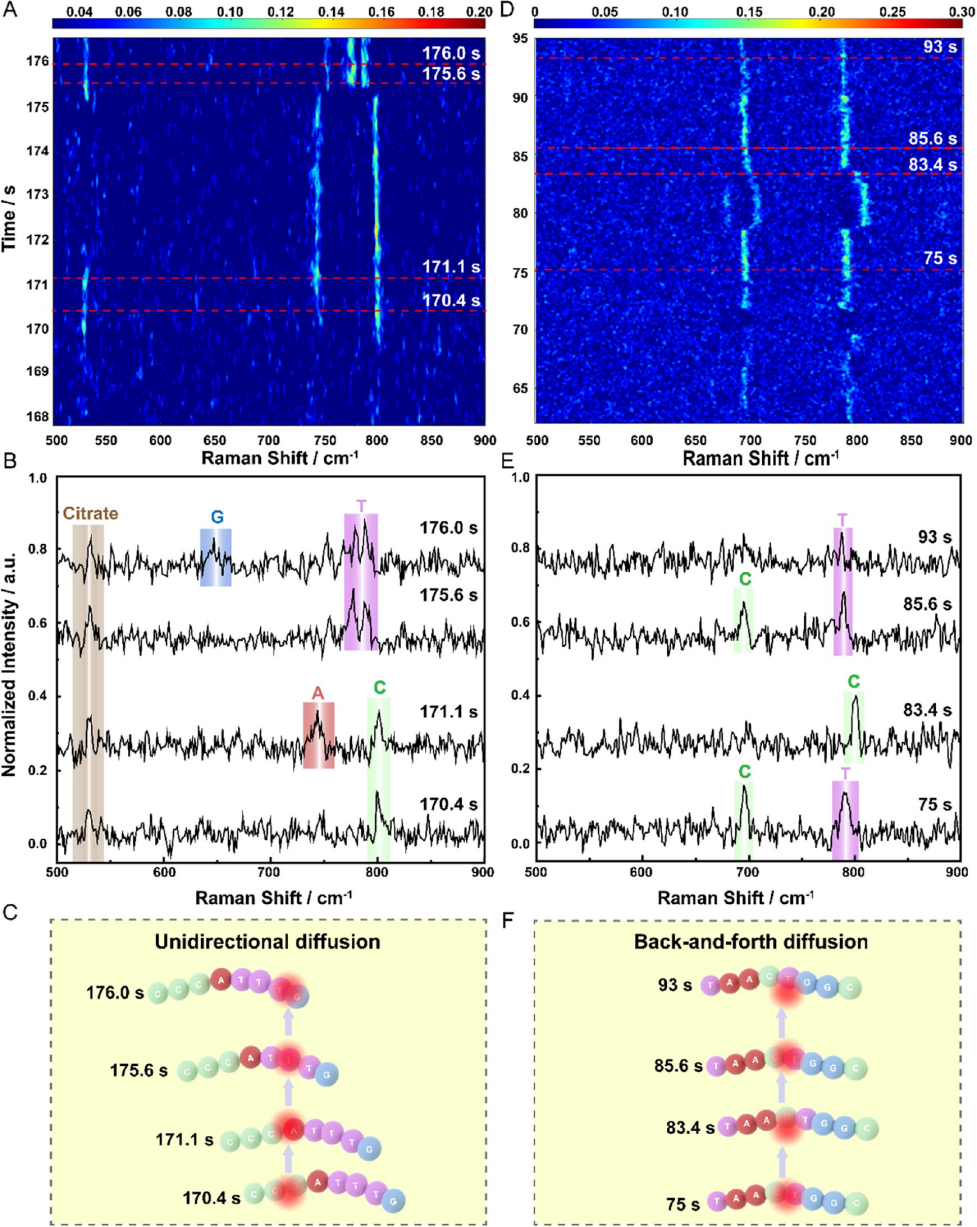
SM-SERS monitoring of the unidirectional diffusion of the 5′-CCCATTTG-3′ and the back-and-forth diffusion of the 5′-TAACTGGC-3′ oligonucleotides. (A) 168–177 s range of time series of 5′-CCCATTTG-3′ oligonucleotide. (B) Peak assignment SERS spectra of different nucleobases of 5′-CCCATTTG-3′ and citrate. (C) A schematic of unidirectional molecule diffusion of different nucleobases of 5′-CCCATTTG-3′ under a plasmonic hot spot. (D) 62–95 s range of time series of 5′-TAACTGGC-3′ oligonucleotide. (E) Peak assignment SERS spectra of different nucleobases of 5′-TAACTGGC-3′. (F) A schematic of back-and-forth molecule diffusion of different nucleobases of 5′-TAACTGGC-3′ under a plasmonic hot spot.

**Table 2:** Corresponding Vibration Mode of the Characteristic Peak of Different Bases

DNA nucleobases	characteristic Raman shift/cm^–1^	vibration mode
adenine	735	ring breath
cytosine	802	ring breath
guanine	655	R6 breath
thymine	781	ring breath

Utilizing SERS time series waterfall data, the ability to dynamically read the sequential sequence of DNA oligonucleotide in a single hot spot was demonstrated. As shown in Figure 4B,C, a distinct peak at ∼802 cm^–1^ appeared at 170.4 s, belonging to the C base, indicating the C base in the hot spot. Shortly thereafter, a peak at ∼735 cm^–1^ appeared at 171.1 s, corresponding to the A base appearing, while the C signal was still detectable. This signal overlap indicated that the A base was spatially adjacent to the C base in the chain and both were simultaneously excited in the hot spot. The subsequent disappearance of A and C base signals occurred at 175.6 s, followed by the emergence of a T base peak (∼781 cm^–1^). It marked a diffusion event where new bases entered the hot spot. At 176.0 s, the appearance of a G base-associated signal (∼647 cm^–1^) alongside T indicated a further sequence progression. Partial diffusion trajectories (C → C/A → T → T/G) revealed unidirectional movement of the sequence through the plasmonic hot spot. The relatively long dwell time of the C base was consistent with its abundance in the DNA oligonucleotide sequence (5′-CCCATTTG-3′), and the overall trend partially reflects known gold-surface affinities of nucleobases (A > C ≥ G > T). In addition, the peak of citrate was also observed at 530 cm^–1^. The continuous citrate peak suggested that the citrate molecule diffused into the hot spot and cooccupied with the DNA oligonucleotide in the hot spot.

To further validate this approach, a second DNA oligonucleotide (5′-TAACTGGC-3′) was analyzed (Figure 4D). In this case, as shown in Figure 4E, the T base (∼790 cm^–1^) and another C-associated peak (∼695 cm^–1^) appeared at 75 s. Then, the T base moved out of the hot spot, and only the C base (∼801 cm^–1^) was detected at 83.4 s. At 85.6 s, the C (∼696 cm^–1^) and T (∼790 cm^–1^) bases moved back to the hot spot. The subsequent disappearance of the C base signal at 93 s was followed by the emergence of a T base peak (∼781 cm^–1^), revealing a partial diffusion trend of C/T → C → C/T → T (Figure 4F). The trend shows a typical back-and-forth molecule diffusion in the hot spot.

Furthermore, DNA oligonucleotide fragments and citrate were applied to assess the size of the hot spot based on the diffusion of DNA oligonucleotide and citrate. Since Particle-in-well sensor is a form of gold nanoparticle-on-mirror (NPoM) systems or previously reported picocavities, their hot spots are typically formed by single or a few random atomic protrusions on the nanoparticle surface.^3,38^ When the nanowell sidewall is close to the nanoparticle, plasmonic coupling between the particle and sidewall generates hot spots at the atomic protrusions, generating extremely strong local electric fields within the nanogap. Brolo et al. reported that such random atomic protrusion on dry nanoparticle surface would fluctuate under laser illumination at the time scale of 100 μs, while the SERS signal fluctuations on dry nanoparticles at 0.01–1 s time scale came from the molecular diffusions.^17^ It means that the SM-SERS signals from the static hot spot in the Particle-in-well sensor at our exposure time scale of 0.1 s were from DNA diffusion through an average hot spot of fast atom fluctuation.

Given that the DNA oligonucleotide contains a sugar-free phosphate backbone (B), additional examples including the O–P–O stretching of the DNA backbone were identified to further support the molecular diffusion behavior of C → A/B → T/B, as reflected in the signal changes observed at different regions of the DNA oligonucleotide chain in the hot spot (Figure S6, Supporting Information). The aromatic rings of the nucleobases have a higher affinity with the gold surface that the SERS bands of the backbone appear occasionally due probably to conformation change during DNA diffusion.^11^ The backbone (∼825 cm^–1^) appeared simultaneously with the nucleobase signal, depending on the conformation of the DNA oligonucleotide on the gold surface.^39^ Therefore, these data led us to assume that the hot spot size could cover 2 DNA nucleobases, 1 sugar–phosphate backbone, and 1 citrate.

To demonstrate the potential of this method for quantitative analysis of the A bases, 2000 spectra were collected from the No. 3 DNA oligonucleotide (5′-TACAAGTAAAG-3′), as shown in Figure 5A,D. In this case, the peak corresponding to the A base (∼735 cm^–1^) appeared together with the citrate peak (∼630 cm^–1^) and backbone band (B band: ∼826 cm^–1^) at 55.3 s, followed by a G base peak (∼655 cm^–1^) at 62.5 s. Both A and G bases were simultaneously excited in the plasmonic hot spot at 63 s, resulting in alternating signals from A and G that persisted for a period. These observations revealed a partial molecule diffusion sequence of A → G → A and G (Figure 5B). Due to DNA nucleobase diffused out of the hot spot or DNA conformation change, the citrate and B band appeared (∼630, ∼826 cm^–1^) at 66 s. Figure 5C shows that the back-and-forth diffusion of the DNA oligonucleotide was in the hot spot. Therefore, the continuous observation of the DNA backbone signals along with characteristic nucleobase peaks, together with previously reported findings,^16,40,41^ indicates that the DNA retained its native structure and did not undergo laser-induced damage in the Particle-in-well sensor.

**Figure 5:**
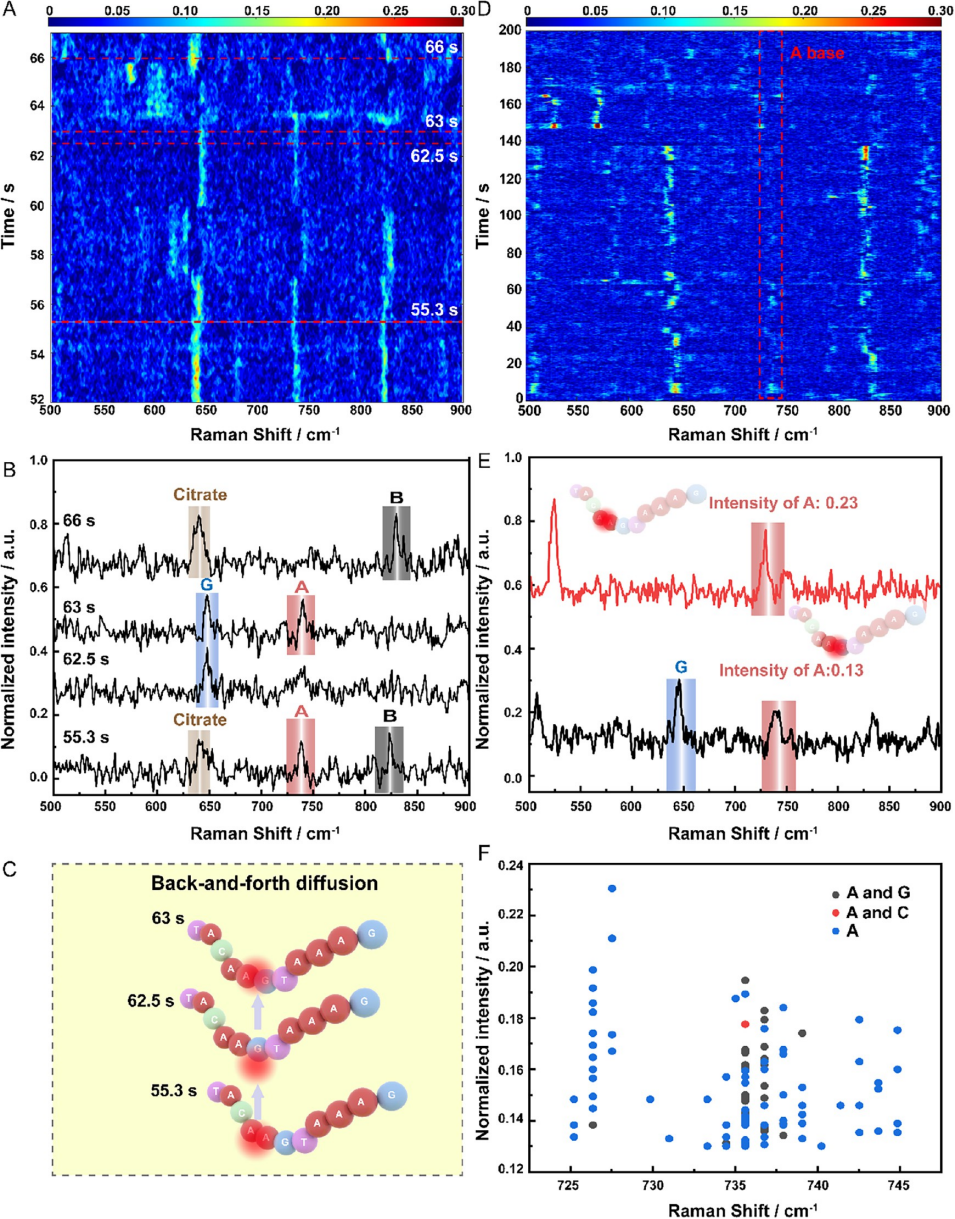
Identification of single nucleobases and quantitative analysis in the 5′-TACAAGTAAAG-3′ oligonucleotide. (A) 52–69 s range of time series of the 5′-TACAAGTAAAG-3′ oligonucleotide. (B) Peak assignment SERS spectra of different nucleobases, citrate, and backbone (B) band. (C) A schematic of back-and-forth molecule diffusion of different nucleobases under a plasmonic hot spot. (D) 2000 time series spectra of the 5′-TACAAGTAAAG-3′ oligonucleotide. (E) Intensity comparison of a spectrum containing A and G base peaks (intensity of A base: ∼0.13) and a spectrum containing only A base peak (intensity of A base: ∼0.23). The inset is a schematic diagram of the corresponding molecular diffusion under the plasmonic hot spot (the inset above shows more than one A base in the hot spot, and the inset below shows one A and one G in the hot spot). (F) Intensity distribution of A base at around 735 cm^–1^ peaks using effective spectra from 2000 time series spectra. (Red dot means the peak intensity of the A base in the spectra with signals of both A and C bases. Black dot means the peak intensity of the A base in the spectra with signals of both A and G bases. Blue dot means the peak intensity of the A base in the spectra only with the signal of A base.).

Due to the high abundance of A bases in the DNA chain, the dynamic movement and diffusion of the oligonucleotide caused varying numbers of A bases to enter the hot spot and different positions of A base in the hot spot, leading to different intensities of A base SERS signals. In Figure 5E, when both A and G bases existed in the hot spot, the peak intensity was different from that when there is only an A base, indicating that different numbers of A bases contribute to the signal. For example, the intensity of the A base peak in the red spectrum (normalized intensity: 0.23) was almost twice that of the black spectrum (normalized intensity: 0.13). This means that more than one A base was in the hot spot. What’s more, after screening 2000 data sets, 105 effective spectra containing A base signals were selected for statistical analysis. The corresponding intensity distribution of A base peak at ∼735 cm^–1^ was extracted from all effective spectra (Figure 5F). It extracted the peak intensity values of A base from all spectra containing A base’s signal. The results showed that A base could be in the hot spot with the G or C base, or it could be in the hot spot alone. Moreover, different combinations and subtle position changes could induce different peak intensities of the A base. These results indicated that this Particle-in-well sensor holds potential to perform quantitative analysis by monitoring the diffusion behavior of DNA oligonucleotide at the single-molecule level.

To emphasize the long-term performance of this Particle-in-well sensor, 10000 SERS spectra of 5′-TACAAGTAAAG-3′ were collected in a 17 min period. As shown in Figure S7, it clearly revealed a three-stage diffusion motion: (1) DNA oligonucleotide diffused into the hot spot within 0–250 s, (2) DNA oligonucleotide diffused out of the hot spot after about 7 min, and (3) DNA oligonucleotide diffused back into the hot spot at around 12.5 min. Here, additional data of 30 and 60 min SM-SERS time series were also collected to further confirm the long-term SERS monitoring performance, as shown in Figure S8. These results further demonstrate the dynamic diffusion behavior of DNA oligonucleotide in the hot spot as monitored by both unidirectional and back-and-forth diffusion during long-term signal acquisition.

### Diffusion Time Measurement by Single-Molecule Raman Correlation Spectroscopy

As the citrate may diffuse into the hot spot together with the DNA oligonucleotides, we applied Raman correlation spectroscopy to the Particle-in-well sensor to calculate their diffusion time on the particle surface in an air environment. We analyzed the average diffusion time of DNA oligonucleotide No. 1 (5′-CCCATTTG-3′) and citrate in the hot spot and further distinguished the signal of single-molecule citrate and DNA oligonucleotide.^42^ The DNA oligonucleotide’s diffusion time was calculated using peaks near 655, 735, 781, and 802 cm^–1^ for each time series data set. Then, the diffusion time of citrate was calculated using signals near 520, 630, 830, and 890 cm^–1^. Five time series plots with each 2000 spectra were applied to the statistical diffusion time overall trend of citrate and DNA oligonucleotide. Correspondingly, there are 20 data sets for citrate and DNA oligonucleotide, respectively, to calculate their diffusion time. For example, one of the five time series waterfall plot of citrate and DNA oligonucleotide is shown in Figure 6A,E, respectively. Time traces of SERS signals at 890, 830, 630, and 520 cm^–1^ of citrate and 802, 781, 735, and 655 cm^–1^ of DNA oligonucleotide from time series plots, such as those in Figure 6B,F, were used to compute an autocorrelation value (Figure 6C,G). The final 20 results of DNA oligonucleotide and citrate are extracted from the autocorrelation plot, as shown in Figure 6D,H, respectively. The function of the kind G­(τ) = ⟨F­(t) F­(t + τ)⟩/⟨F­(t)⟩^2^, where F­(t) is the Raman time trace, τ is the lag time, and ⟨⟩ indicates time averaging. The diffusion times were extracted by fitting the autocorrelation function with a two-dimensional (2D) Gaussian diffusion model: G­(τ) = a + 1/N­(1 + τ/τ_D_),^43^ where N is the average number of particles in the detection volume, a represents a baseline or offset, and τ_D_ is the average diffusion time of the particles through the detection volume. Before analyte substitution, according to the fitting results from the autocorrelation function of SERS data, 80% of the diffusion time of citrate was below 2.5 s in Figure 6D. After DNA oligonucleotide substitutes the citrate, 60% of the diffusion time of the DNA oligonucleotide was below 2.5 s due to the low abundance of A and G bases (red and black columns) in the DNA oligonucleotide chain in Figure 6H. However, 87.5% of the diffusion time of C and T bases was above 2.5 s due to the high abundance of C and T bases in the DNA oligonucleotide chain (green and yellow columns). The average diffusion times of citrate and DNA oligonucleotide are 1.37 and 4.16 s, respectively.

**Figure 6:**
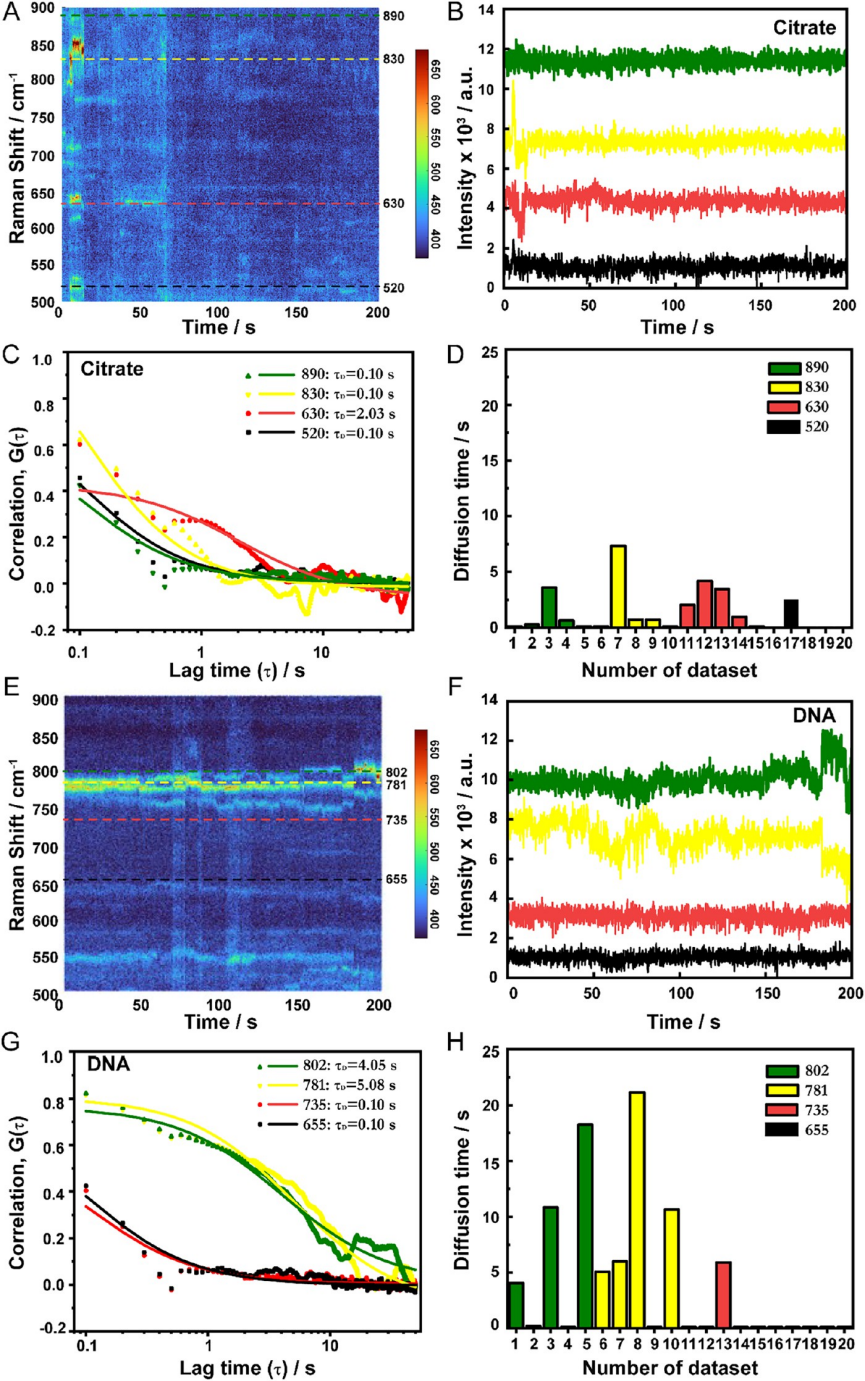
Diffusion time statistics between citrate and DNA oligonucleotide SERS signal. (A) A waterfall plot of the SERS time series of citrate. (B) The SERS time traces of citrate at 520 (black), 630 (red), 830 (yellow), and 890 cm^–1^ (green). (C) Autocorrelation function (scatter) associated with the SERS time trace, and it fits a 2D diffusion model (line) of citrate. τ_D_ of citrate at 520, 630, 830, and 890 cm^–1^ are 0.1, 2.03, 0.1, and 0.1 s, respectively. (D) Statistical results of diffusion time for 20 data sets of citrate. (E) One SERS time series spectra of a DNA oligonucleotide. (F) A waterfall plot of SERS time series of DNA oligonucleotide at near 655 (black), 735 (red), 781 (yellow), and 802 cm^–1^ (green). (G) Autocorrelation function (scatter) associated with the SERS time trace, and it fits a 2D diffusion model (line) of a DNA oligonucleotide. τ_D_ of DNA oligonucleotide at 655, 735, 781, and 802 cm^–1^ are 0.1, 0.1, 5.08, and 4.05 s, respectively. (H) Statistical results of diffusion time for 20 data sets of DNA oligonucleotide.

It is well known that if no external force acts on a DNA molecule, then the molecule will maintain its original state. However, under hot spots due to the plasmonic force, the DNA molecular chain undergoes multiple conformational distortions. Correspondingly, the conformation and position of citrate in the hot spot also change. The resulting conformational transitions are characterized by a two-step distortion (a drop between 0.1 and 1 of lag time).^44^ Overall, the average diffusion time of the DNA oligonucleotide was longer than that of citrate. The DNA oligonucleotide diffused more slowly than citrate on the gold surface, which may be due to the longer contour length, higher molecular weight, and the greater gold–DNA affinity mentioned earlier.^26,44,45^ The calculated results and SERS signal indicated that due to the rapid diffusion speed of citrate, the SERS signal of citrate appeared for a short duration and did not last long. However, after the DNA oligonucleotide substitutes citrate, the signal from the DNA oligonucleotide exhibited fluctuations and persistence throughout the testing process. This is also one of the characteristics used to assess the purity of the DNA oligonucleotide signal. Overall, different diffusion times can further validate the distinction between citrate and DNA oligonucleotide signal, as well as prove that their single-molecule diffusion behaviors are different. The SERS characteristic peaks were used to calculate the molecular diffusion trend and time under laser irradiation, proving the feasibility of this approach in the field of single-molecule dynamics research and analysis.

## Conclusion

According to all of our currently available results, the plasmonic hot spot only covers the neighboring bases that are one or two bases in most situations, which shows the potential for sequencing if unidirectional movement of the molecule is guaranteed. If additional nucleobases appear periodically or variously, the measurement or technique can be considered dynamic and suitable for DNA or peptide sequencing. In addition, a certain degree of quantitative analysis can be achieved by SM-SERS spectra to determine the abundance of bases in the DNA chain. However, random diffusion of a single molecule in the Particle-in-well sensor is monitored, and the direction of molecular motion in the hot spot is not unique. Currently, due to the lack of molecule manipulation technology in the Particle-in-well sensor, single-molecule sequencing cannot be achieved at this moment. However, our results have demonstrated that due to the advantages of static hot spot, long-term SM-SERS monitoring of single DNA base readout, and Raman correlation spectroscopy methodology, it has great potential to be applicable to single-molecule DNA, RNA, or peptide sequencing when the molecule diffusion direction can be manipulated.

In summary, the entrapment of gold nanoparticles within a nanowell array has established a powerful Particle-in-well sensor, providing strong plasmonic enhancement and creating small hot spots for single-molecule SERS detection and diffusion monitoring. As evidenced by the BIASERS observations, the successful detection of a single molecule confirms the single-molecule sensitivity of the sensor. In addition, the adsorption of a DNA oligonucleotide chain onto the surface of these gold nanoparticles has further validated the sensor’s effectiveness in single-molecule dynamic monitoring. This approach not only allows the identification of individual nucleobases but also enables the real-time monitoring of oligonucleotide diffusion (sequence: 5′-CCCATTTG-3′, 5′-TAACTGGC-3′, and 5′-TACAAGTAAAG-3′) across the surface of the gold nanoparticles and monitoring of the base abundance. With Raman correlation spectroscopy, the SM-SERS signals can also be used to calculate the diffusion times of different DNA bases as well as citrates on the nanoparticle surface. In conclusion, the Particle-in-well sensor represents a powerful method for advancing research in single-molecule diffusion monitoring at the nanoscale and offers profound insights into single-molecule sequencing by using DNA oligonucleotides as a model molecule.

## Methods

### Materials

Nonfunctionalized 50 nm gold nanoparticles (AuNPs) from Sigma (753645–25 ML, concentration of 3.5 × 10^10^ particles/mL) in 0.1 mM PBS citrate stabilized, reactant free. Dry DNA oligonucleotides (5′-CCCATTTG-3′, 5′-TAACTGGC-3′, and 5′-TACAAGTAAAG-3′) were purchased from Sigma. Silicon wafers with 100 nm Si_3_N_4_ membranes coated on the surface were purchased from MicroChemicals GmbH (WNA4 0525 155B 1314 S102, prime Si+ Si_3_N_4_ 4 in.).

### Apparatuses and Software

The SERS spectra were detected by a ThermoFisher DXR2xi Raman Imaging Microscope and collected by Andor Solis. The nanowells were prepared by Focused Ion Beam. The SEM images of nanowells were taken by Sigma HD VP FE-SEM. The gold layer was sputtered by a Q150T ES Sputter coater. The entrapment was conducted by a power supply, a three-dimensional (3D)-printed case, a PID controller, and a Peltier heater. The data was analyzed by MATLAB R2023a. The size of dynamic light scattering was acquired by a Zetasizer Nano.

### Fabrication of the Nanowell Arrays by FIB

The nanowells were fabricated by an FIE Helios FIB. 100 nm gold was sputtered on the purchased wafer containing 100 nm Si_3_N_4_ on p-type Si 500 μm. Then, the nanowells were fabricated by using FIB, and the estimated depth of the wells is around 100 nm to penetrate only the gold (100 nm) layer.

### Attachment of Submonolayers and Single Molecules on the Gold Nanoparticles

To prepare analyte-loaded (submonolayer of pyridine and pyridine-d5, and 10% coverage of 5′-CCCATTTG-3′, 5′-TAACTGGC-3′, and 5′-TACAAGTAAAG-3′) AuNP colloid, 300 μL of AuNP (50 nm) stock was dispersed in 400 μL of 5 vol % 1× PBS (pH 7.5–7.6) and 100 μL of analyte solution was added to reach the desired concentration, resulting in 800 μL of final volume with 1.3 × 10^10^ AuNP/mL. The dispersion was 10× diluted in order to match the requirements of the capillary assembly (1.3 × 10^9^ AuNP/mL). After mixing, the samples were stored at 4 °C for 2 days.

### Capillary-Assisted Particle Assembly (CAPA)

40 μL of molecule-loaded AuNPs colloidal was confined between the nanowell chip and a glass slide. Molecule-loaded AuNPs were pulled through the nanowell chip. Solvent evaporated from the receding solvent–vapor interface, and evaporation was accelerated by substrate heating at 30 °C, inducing convection that dragged the molecule-loaded AuNPs from the bulk to the surface of the suspension with a 1.5–3 μm/s dragging speed. The receding contact line dragged the accumulation zone across the substrate, causing the nanoparticles to leave the colloidal meniscus and selectively fill the matching nanowells. This indicates successful nanoparticle assembly.

### AFM Measurements

The topography of the nanowells was studied by using atomic force microscopy (AFM). The AFM measurements were performed using the Nanosurf FlexAFM system, equipped with a C3000i Controller (Nanosurf AG, Switzerland). The imaging was done in tapping mode using a Dyn190Al AFM probe with 190 kHz nominal resonant frequency and 48 N/m force constant (Nanosurf AG, Switzerland).

### SERS Measurements

The oligonucleotide chains were absorbed on the surface of the AuNPs for 48 h. Then, the particles can be entrapped in the nanowell. SERS measurements were carried out on selected nanoarrays. The spectra were collected with a 50× objective, λ = 785 nm, laser power of 18–22 mW, exposure time of 0.1 s, full range resolution grid, and 50 μm slit. The time series were collected from the Andor Solis system to acquire 2000 spectra for each time series figure.

### Data Preprocessed and Peak Assignment

The SERS spectra need to be preprocessed before peak assignment by several procedures: (1) clipping, (2) cosmic ray removal, (3) normalization, and (4) background removal. The Raman shift of those spectra was clipped to the interested area of 500–900 cm^–1^. Removing the cosmic ray was applied to the raw SERS spectra using a custom function “remove_cosmic_rays” with a defined threshold to eliminate sharp, noise-induced peaks that could distort the spectral data. The intensity values of the spectra were normalized using Min-Max scaling to rescale the data between 0 and 1. The background removal was used to prevent the impact of baseline and background drift. Subsequently, the SERS spectra were obtained with peak assignment with “findpeak” function in MATLAB. The database has been built according to several references. The peak can be assigned to different bases by defining the peak width, height, prominence, and peak shift range.

## Supplementary Material



## Data Availability

The data that support the findings of this study are available from the corresponding author upon reasonable request.

## Notes

Dimension of the nanowell by AFM; the particle size of AuNPs; DNA-loaded AuNP by DLS; the top-view SEM image of the DNA-loaded AuNP immediately trapped in nanowell and after testing for 40 min and storage for 6 months; substitution of citrates by pyridine and pyridine-d5 on the AuNPs; characteristic fwhm of C and T bases; SM-SERS monitoring of the unidirectional diffusion of the 5′-CCCATTTG-3′; long-term monitoring of the unidirectional diffusion and the back-and-forth diffusion of the 5′-TACAAGTAAAG-3′; long-term collected SM-SERS spectra of 30 min test and 60 min test (PDF)

## #

Deceased August 2, 2024.
