# Supplementary material for: Single-Molecule Monitoring of Nucleic Acid Dynamics Using Raman Correlation Spectroscopy in Plasmonic Nanowells
Source: ACS Nano. 2025 Oct 14;19(42):37241–54. doi: 10.1021/acsnano.5c12000 (PMC12574212; doi:10.1021/acsnano.5c12000)
Supplement: Supplementary file 1 [file nn5c12000_si_001.pdf]

Supporting information

Single-Molecule Monitoring of Nucleic Acid Dynamics Using Raman Correlation Spectroscopy in Plasmonic Nanowells

*Peilin Xin[1,3], Yingqi Zhao[1,3], Yuge Liang[1,3], Mulusew W. Yaltaye[2,3], Aliaksandr Hubarevich[4], Viktorija Pankratova[5], Shubo Wang[5], Jian-An Huang[1,2,3]**

[1] Research Unit of Health Sciences and Technology, Faculty of Medicine, University of Oulu, Aapistie 5 A, 90220 Oulu, Finland.

[2] Research Unit of Disease Networks, Faculty of Biochemistry and Molecular Medicine, University of Oulu, Aapistie 5 A, 90220 Oulu, Finland.

[3] Biocenter Oulu, University of Oulu, Aapistie 5 A, 90220 Oulu, Finland.

[4] Istituto Italiano di Tecnologia, Via Morego 30, 16163, Genova, Italy

[5] Nano and Molecular Systems Research Unit, Faculty of Science, University of Oulu, Pentti Kaiteran katu 1, 90570 Oulu, Finland

*Email: jianan.huang@oulu.fi

Content

Figure S1. The dimension of the nanowell is characterized by AFM...3

Figure S2. The particle size of AuNPs, CCCATTTG@AuNPs and TAACTGGC@AuNPs by Dynamic Light Scattering (DLS). 3

Figure S3. The top view SEM image of the DNA-loaded AuNP immediately trapped in nanowell (A) and after testing for 40 minutes and storage for 6 months (B). 4

Figure S4. Substitution of citrates by pyridine and pyridine-d5 on the AuNPs characterized by the Particle-in-well. (A) The frequency histogram of citrate by bare AuNPs trapped in the nanowell. In the range of 980-1000 cm^{-1}, there are almost no peaks of citrate, and most of the frequencies are close to 0, although in the region of 950 to 960 cm^{-1} with a frequency less than 0.01. (B) The frequency histogram of pyridine and pyridine-d5 absorbed on the AuNPs that were trapped in the nanowell. For pyridine, the region from 980 to 1000 cm^{-1} shows a continuous, higher frequency than that of citrate. The same result is shown for pyridine-d5 from 950 to 970 cm^{-1}. The different distributions between citrates and pyridine, pyridine-d5 confirmed the substitution of citrates..4

Figure S5. Characteristic peak width distribution (FWHM) of C and T base. (A) The FWHM distribution of C nucleobase at around 802 cm^{-1}. (B) The FWHM distribution of T nucleobase at around 781 cm^{-1}. 5

Figure S6. SM-SERS monitoring of the unidirectional diffusion of the 5′-CCCATTTG-3′ (C→A/B→T/B). (A) 170-200 s range of time series of 5′-CCCATTTG-3′ oligonucleotide. (B) Peak assignment SERS spectra of different nucleobases of 5′-CCCATTTG-3′. The corresponding SERS spectra of C, A/B and T/B at 191 s, 193.9 s and 198.5 s, respectively. 5

Figure S7. SM-SERS long-term monitoring of the unidirectional diffusion and the back-and-forth diffusion of the 5′-TACAAGTAAAG-3′ oligonucleotide. (A) 17 min of time series of 5′-TACAAGTAAAG-3′ oligonucleotide with three-stage of DNA oligonucleotide into, out of and back to the hot spot. (B) Time series and peak assignment SERS spectra of 5′-TACAAGTAAAG-3′ and citrate from 175 to 190 s and 30 to 50 s. (C) Schematic of unidirectional molecule diffusion of different nucleobases of 5′-TACAAGTAAAG-3′ under plasmonic hot spot. (D) Time series and peak assignment SERS spectra of 5′-TACAAGTAAAG-3′ and citrate from 190 to 230 s. (E) Schematic of back-and-forth molecule diffusion of different nucleobases of 5′-TACAAGTAAAG-3′ under plasmonic hot spot. 7

Figure S8. The long-term collected SM-SERS spectra of 30 min-test (A) and 60 min-test (B), respectively. .7

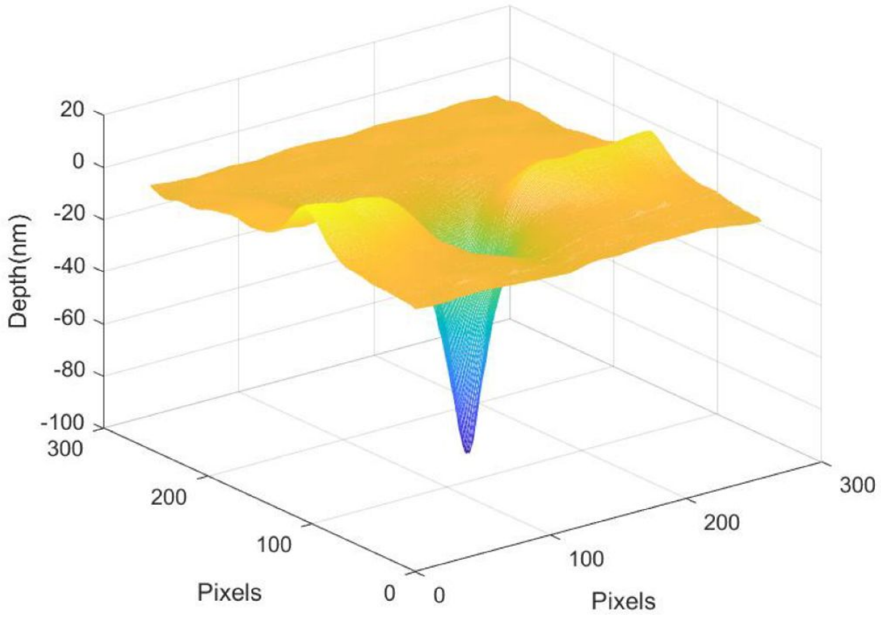

Figure S1. *The dimension of the nanowell is characterized by AFM.*

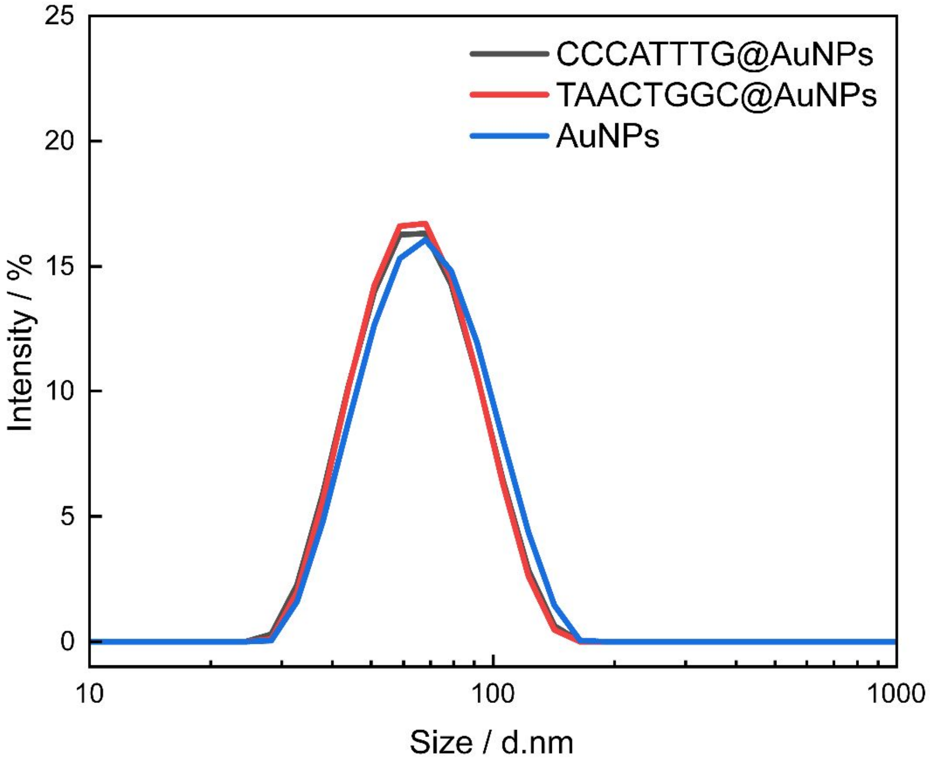

Figure S2. *The particle size of AuNPs, CCCATTTG@AuNPs and TAACTGGC@AuNPs by Dynamic Light Scattering (DLS).*

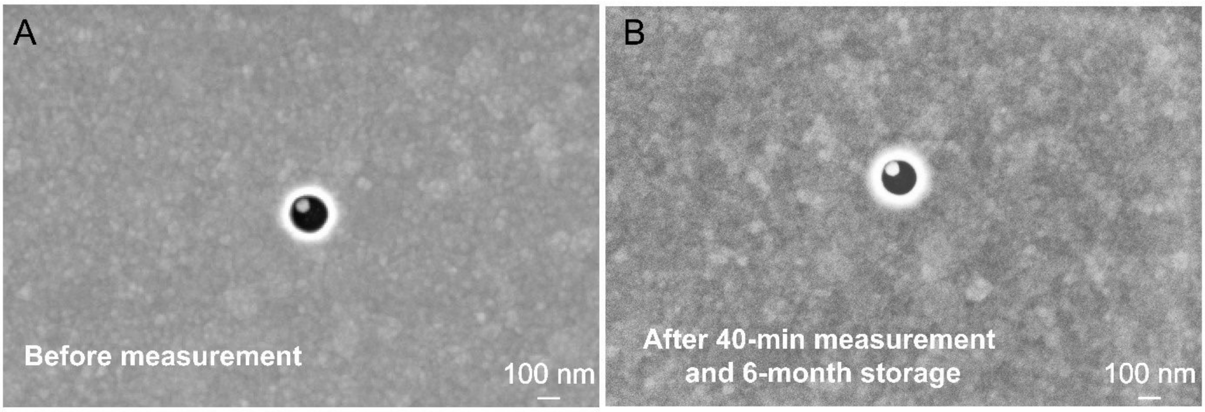

Figure S3. *The top view SEM image of the DNA-loaded AuNP immediately trapped in nanowell (A) and after testing for 40 minutes and storage for 6 months (B).*

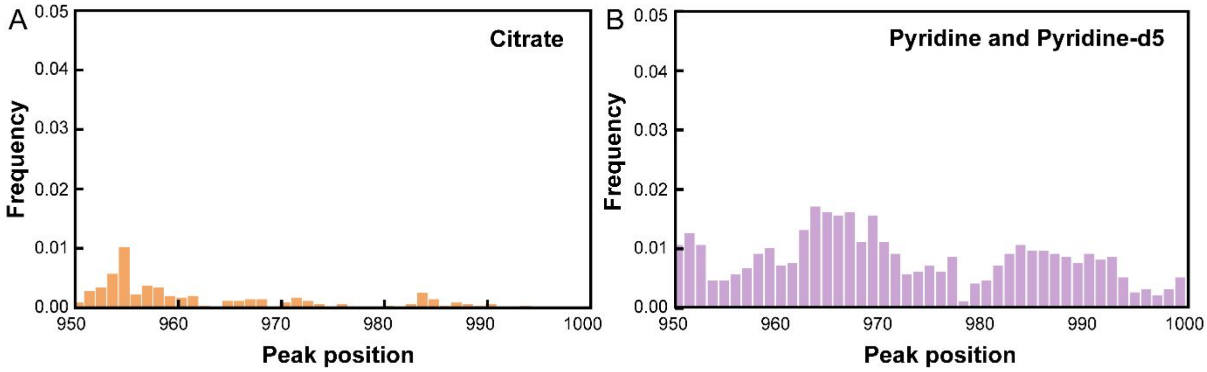

Figure S4. *Substitution of citrates by pyridine and pyridine-d5 on the AuNPs characterized by the Particle-in-well. (A) The frequency histogram of citrate by bare AuNPs trapped in the nanowell. In the range of 980-1000 cm*$^{-1}$*, there are almost no peaks of citrate, and most of the frequencies are close to 0, although in the region of 950 to 960 cm*$^{-1}$ *with a frequency less than 0.01. (B) The frequency histogram of pyridine and pyridine-d5 absorbed on the AuNPs that were trapped in the nanowell. For pyridine, the region from 980 to 1000 cm*$^{-1}$ *shows a continuous, higher frequency than that of citrate. The same result is shown for pyridine-d5 from 950 to 970 cm*$^{-1}$*. The different distributions between citrates and pyridine, pyridine-d5 confirmed the substitution of citrates.*

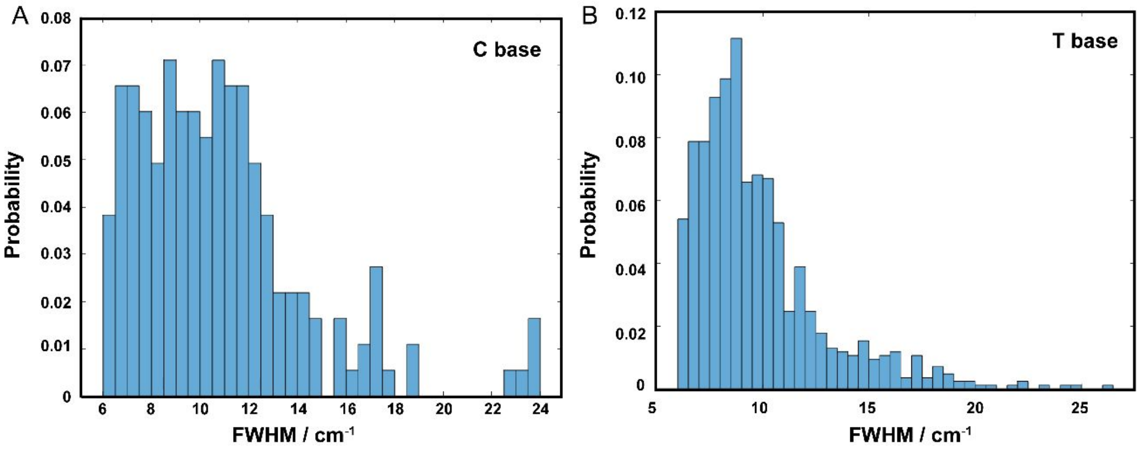

Figure S5. *Characteristic peak width distribution (FWHM) of C and T base. (A) The FWHM distribution of C nucleobase at around 802 cm*$^{-1}$*. (B) The FWHM distribution of T nucleobase at around 781 cm*$^{-1}$*.*

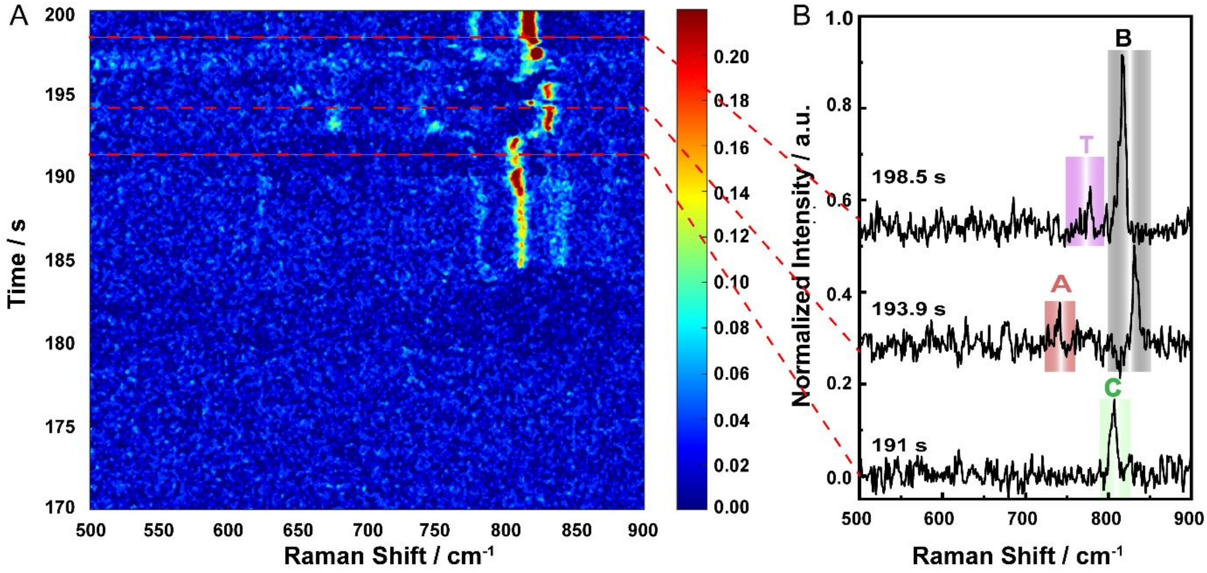

Figure S6. *SM-SERS monitoring of the unidirectional diffusion of the 5′-CCCATTTG-3′ (C→A/B→ T/B). (A) 170-200 s range of time series of 5′-CCCATTTG-3′ oligonucleotide. (B) Peak assignment SERS spectra of different nucleobases of 5′-CCCATTTG-3′. The corresponding SERS spectra of C, A/B and T/B at 191 s, 193.9 s and 198.5 s, respectively.*

Supplementary Note to Figure S7:

To further validate the long-term performance of this particle-in-well sensor, 10000 SERS spectra of 5′-TACAAGTAAAG-3′ were collected in 17 minutes. It showed three-stages diffusion motion in Figure S7A: 1) DNA oligonucleotide diffused into the hot spot; 2) DNA oligonucleotide diffused out of the hot spot, and 3) DNA oligonucleotide diffused back into the hot spot. In stage 1, two regions were extracted to further analyse the diffusion motion. As shown in Figure S7B: typical unidirectional

diffusion, with blinking of C (~793 cm^{-1}), T (~786 cm^{-1}), A (~730 cm^{-1}) base appeared accompanied by the phased appearance of citrate (~545 cm^{-1}). What's more, the backbone also observed at ~825 cm^{-1}. Figure S7C revealed a partial diffusion trend of C→A→T. However, back-and-forth diffusion in Figure S7D, with blinking of T base (~785 cm^{-1}) and continuous appearance of A (~731 cm^{-1}) and C (~796 cm^{-1}) base in the range of 190 to 230 s. A base persisted for approximately 7 and 10 s, respectively. C base persisted for approximately 10 s. It presented the segment of T, A and C base wandering in the hot spot with a trend of A→T→A→C/A in Figure S7E. In stage 2, almost all of signals disappeared for around 7 minutes. It demonstrated the DNA oligonucleotide was not located in the hot spot for stage 2. Then, the DNA oligonucleotide signal was back and blinking in stage 3. According to these results, it further proved the dynamic diffusion behaviour of DNA oligonucleotide in the hot spot as monitored by both unidirectional and back-and-forth diffusion during long-term signal acquisition.

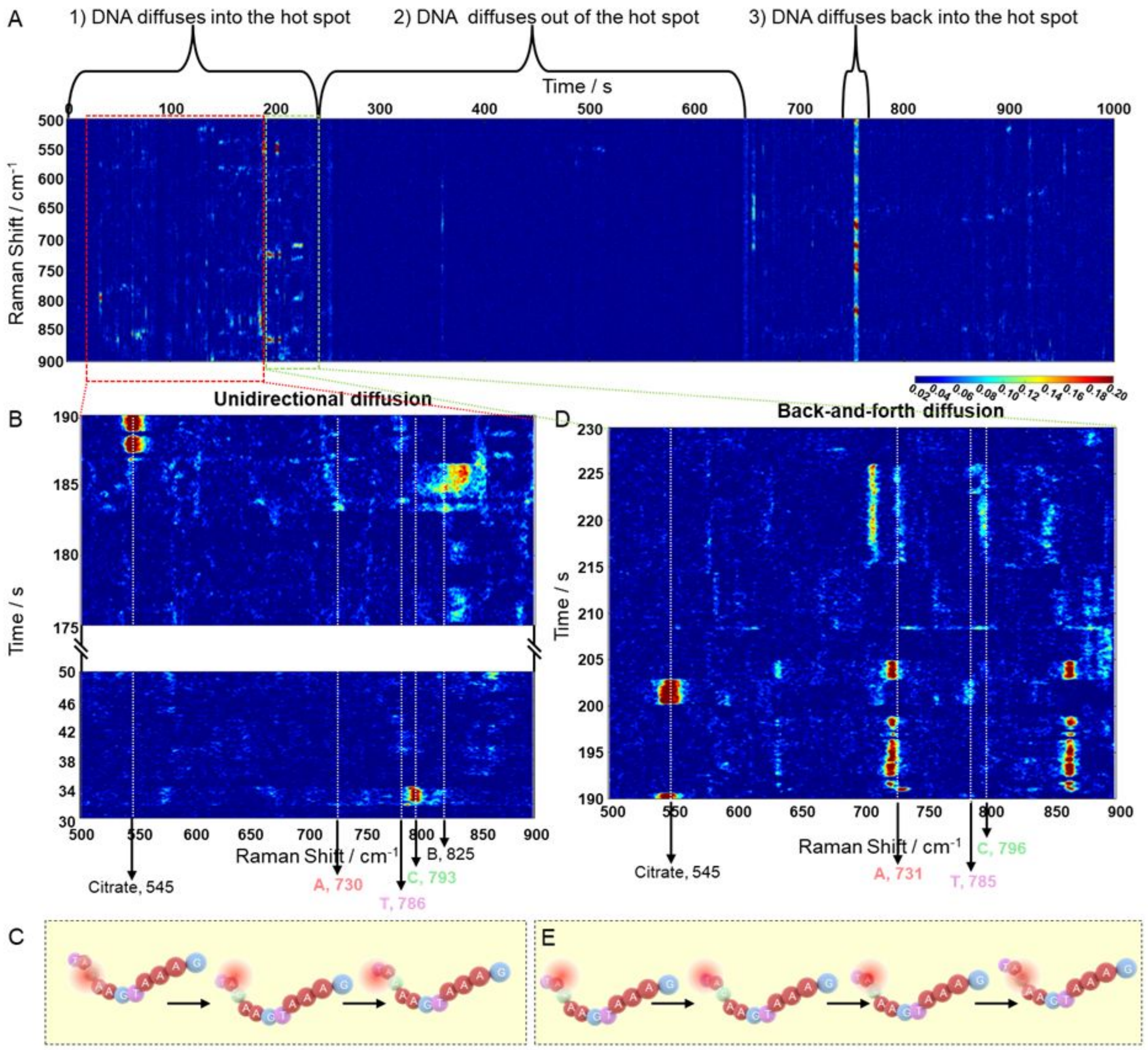

Figure S7. *SM-SERS long-term monitoring of the unidirectional diffusion and the back-and-forth diffusion of the 5'-TACAAGTAAAG-3' oligonucleotide. (A) 17 min of time series of 5'-TACAAGTAAAG-3' oligonucleotide with three-stage of DNA oligonucleotide into, out of and back to the hot spot. (B) Time series and peak assignment SERS spectra of 5'-TACAAGTAAAG-3' and citrate from 175 to 190 s and 30 to 50 s. (C) Schematic of unidirectional molecule diffusion of different nucleobases of 5'-TACAAGTAAAG-3' under plasmonic hot spot. (D) Time series and peak assignment SERS spectra of 5'-TACAAGTAAAG-3' and citrate from 190 to 230 s. (E) Schematic of back-and-forth molecule diffusion of different nucleobases of 5'-TACAAGTAAAG-3' under plasmonic hot spot.*

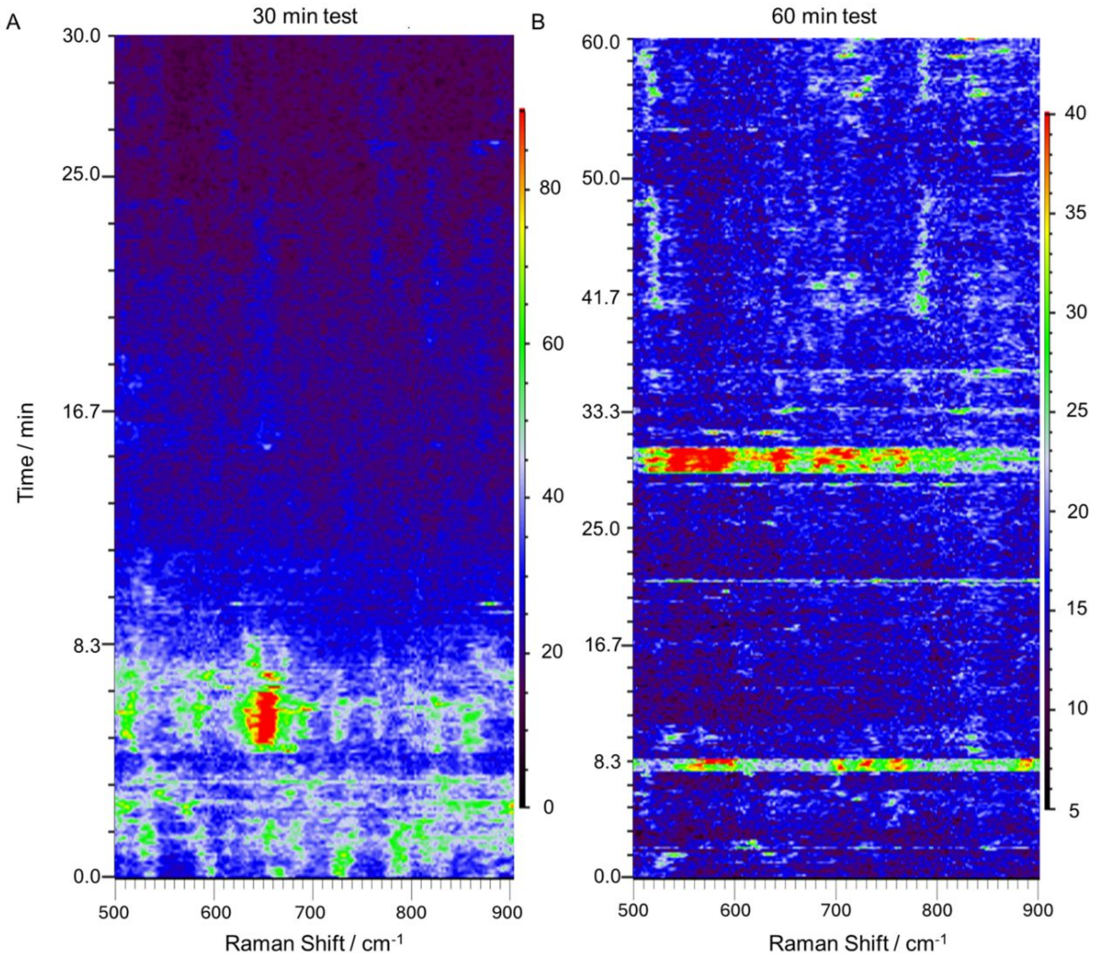

***Figure S8**. The long-term collected SM-SERS spectra of 30 min-test (A) and 60 min-test (B), respectively.*
